# Quantification of sterol-specific response in human macrophages using automated imaged-based analysis

**DOI:** 10.1186/s12944-017-0629-9

**Published:** 2017-12-13

**Authors:** Deborah L. Gater, Namareq Widatalla, Kinza Islam, Maryam AlRaeesi, Jeremy C. M. Teo, Yanthe E. Pearson

**Affiliations:** 1 Department of Chemistry, Khalifa University, P.O. Box 127788, Abu Dhabi, United Arab Emirates; 2 Department of Biomedical Engineering, Khalifa University, P.O. Box 127788, Abu Dhabi, United Arab Emirates; 3 New York University, P.O. Box 129188, Abu Dhabi, United Arab Emirates; 4 Center for Genomics and Systems Biology, New York University Abu Dhabi, Abu Dhabi, United Arab Emirates

**Keywords:** Cholesterol, Ergosterol, Foam cell, Image processing, Lipid droplet, Lysophosphatidylcholine, THP-1, Vesicle, Watershedding

## Abstract

**Background:**

The transformation of normal macrophage cells into lipid-laden foam cells is an important step in the progression of atherosclerosis. One major contributor to foam cell formation in vivo is the intracellular accumulation of cholesterol.

**Methods:**

Here, we report the effects of various combinations of low-density lipoprotein, sterols, lipids and other factors on human macrophages, using an automated image analysis program to quantitatively compare single cell properties, such as cell size and lipid content, in different conditions.

**Results:**

We observed that the addition of cholesterol caused an increase in average cell lipid content across a range of conditions. All of the sterol-lipid mixtures examined were capable of inducing increases in average cell lipid content, with variations in the distribution of the response, in cytotoxicity and in how the sterol-lipid combination interacted with other activating factors. For example, cholesterol and lipopolysaccharide acted synergistically to increase cell lipid content while also increasing cell survival compared with the addition of lipopolysaccharide alone. Additionally, ergosterol and cholesteryl hemisuccinate caused similar increases in lipid content but also exhibited considerably greater cytotoxicity than cholesterol.

**Conclusions:**

The use of automated image analysis enables us to assess not only changes in average cell size and content, but also to rapidly and automatically compare population distributions based on simple fluorescence images. Our observations add to increasing understanding of the complex and multifactorial nature of foam-cell formation and provide a novel approach to assessing the heterogeneity of macrophage response to a variety of factors.

**Electronic supplementary material:**

The online version of this article (10.1186/s12944-017-0629-9) contains supplementary material, which is available to authorized users.

## Background

Despite many decades of medical research and public health activity, cardiovascular disease (CVD) remains one of the leading causes of death world-wide, with underlying atherosclerosis being an important contributing factor in CVD morbidity and mortality rates, in both the developed and the developing world [1]. The role of macrophages in the pathogenesis of atherosclerotic plaques is complex, and has been well reviewed elsewhere [2, 3]. In brief, circulating monocytes are first recruited to localized sites of damage or inflammation on the artery wall by an accumulation of low-density lipoprotein (LDL) and by apolipoprotein-B (ApoB) -containing particles. Secondly, these cells penetrate the intima and differentiate first to macrophages, and then to lipid-laden foam cells, following activation by an array of inflammatory factors. Finally, the foam cells rupture, depositing yet more lipids and inflammatory factors into the immediate area within the artery wall and contributing to a detrimental positive feedback loop that may ultimately result in plaque formation. In this work, we are particularly interested in investigating the parameters contributing to the second of these steps, during which macrophages are transformed into foam cells, and in applying a novel computational method to assess the heterogeneity of the cellular response to a variety of factors.

The conversion of macrophages into foam cells involves the disruption of the cells’ native cholesterol processing pathways [4, 5]. The uptake of cholesterol (predominantly in the form of cholesterol esters encapsulated in LDL) is accelerated by membrane proteins, including scavenger receptors scavenger receptor A (SRA), CD36 and CD68, resulting in the internalization of cholesterol esters that are broken down to free cholesterol in lysosomes [4, 5]. As this exogenous cholesterol accumulates within the cell, the endogenous cholesterol synthesis pathway – through the sterol regulatory element-binding proteins (SREBPs) – is suppressed [6]. In order to be eliminated from the cell (usually as high-density lipoprotein via the reverse cholesterol transport pathway), the accumulated free cholesterol must be re-esterified by enzymes such as sterol O-acyltransferase (SOAT, also known as acyl-CoA cholesterol acyltransferase – ACAT) in a process regulated by the liver X receptor (LXR) and the retinoid X receptor (RXR) [7, 8]. In a competing pathway, cholesterol esters may be again broken down to free cholesterol by enzymes such as hormone sensitive lipase [4, 5]. If exogenous cholesterol accumulates too quickly within a cell, it can overwhelm the LXR-regulated reverse transport pathway and result in the buildup of large quantities of cholesterol and associated lipids – potentially resulting in excessive lipid droplet formation, upregulation of a number of inflammatory factors and ultimately cell death [9].

Here, we have extended previous work by others [10, 11], by examining the susceptibility of monocyte (human THP-1) derived macrophages to uptake large quantities of lipid and cholesterol particles and vesicles (such as accumulate in more advanced fatty streaks [12–14]), and the subsequent effect on cell survival, cell area, cell eccentricity and relative lipid content of cells. The basic lipid particles used comprised cholesterol and lysophosphatidylcholine (LPC, a compound that is both a constituent of oxLDL [15] and that has raised levels in plasma of patients with CVD [16]). We have also substituted the cholesterol within these particles with either the fungal sterol ergosterol or the head group-modified cholesterol derivative cholesteryl hemisuccinate (CHS). Finally, we have examined the contributions of serum, of co-addition of LDL or macrophage inflammatory activator, lipopolysaccharide (LPS), and of pre-incubation with anti-ApoB-antibodies on macrophage response to these lipid particles.

One interesting aspect of studies of foam cell formation relates to the inherent heterogeneity of the macrophage population both in vitro and in atherosclerotic plaques [17–19]. These observations motivate studies that are able to report single-cell results for large numbers of cells, in order to assess the extent and magnitude of variations within populations of cells exposed to the same stimuli. However, the ability and accessibility to measure cellular phenotype both accurately and efficiently, with cell-to-cell consistency in measurement metrics, such as shape, size and staining, from hundreds of digital images, is often the bottleneck for clinicians and experimentalists alike, yet remains essential for thorough analysis, both qualitative and quantitative. While many segmentation methods have been developed, experiment-specific tailoring of such schemes remains a necessary component for consistently objective automated cell boundary detection [20–22].

To date, both manual and semi-automated ‘per-cell’ measurements are still commonly used; however, the complexity of cell conformations (per cell and per image) presents a major barrier, preventing effective determination of unbiased and consistent cell boundaries, and introduces measurement error that propagates through subsequent assessment of cellular parameters and statistical analysis.

To address these issues, we present and discuss the output of a newly developed, customized image-processing method, designed to identify individual cells via boundary detection (from populations and clusters) by applying watershed image segmentation, implemented in MATLAB software (Mathworks Inc.) (see also reference [23]), to quickly extract and analyze data from bright-field and fluorescence images to provide single-cell properties, such as area, extent of staining and intracellular heterogeneity as well as eccentricity, for over one thousand cells in 25 different chemical environments.

## Methods

### Cell culture

THP-1 monocytes (ATCC® TIB-202™) were maintained at 37 °C in 5% CO_2_ and cultured according to instructions. THP-1 media consists of RPMI 1640 (SH30255.01, Hyclone), supplemented with 10% fetal bovine serum (FBS, F2442, Sigma) and 1% penicillin-streptomycin (P4333, Sigma). Only passage 15 THP-1 was used for experiments. THP-1 cells were selected for this study to allow for direct comparison with previous studies in the same cell-line, and to avoid the inherent variation present in primary cell populations – as we wished to examine how different conditions affected cell populations.

### Differentiating protocol

A 3-day treatment with Phorbol 12-myristate 13-acetate (PMA, 5 ng/mL, P1585, Sigma) was used to stably differentiate suspension cultured THP-1 into macrophages without causing undesirable gene upregulation that overwhelmed gene expression induced by weak stimuli [24]. Differentiation was confirmed through bright field microscopy (Axio Zeiss Observer Z.1, magnifications 10× and 20×), cell adherence, elongation and spreading are hallmarks of macrophages. Cells were then trypsinized (0.05% Trypsin-EDTA, 10 min, Sigma), resuspended in FBS free THP-1 media, and plated at 2.1 × 10^4^ cells/well (SIAL0596, Sigma). Cells were left to recover in PMA-free medium for 1 day prior to further use.

### Cellular lipid accumulation protocol

Five different conditions were studied: (1) control/vehicle (PBS only, 10 μL) – termed “PBS”; (2) 16:0 lysophosphatidylcholine (LPC, Avanti Polar Lipids) in PBS (10 μl of 6 μmol/ mL) – termed “LPC”; (3) a 1:1 mixture of LPC and cholesterol (Sigma) in PBS (10 μl of 6 μmol/ mL) – termed “Chol”; (4) a 1:1 mixture of LPC and ergosterol (Sigma) in PBS (10 μl of 6 μmol/ mL) – termed “Erg”; and (5) a 1:1 mixture of LPC and cholesteryl hemisuccinate (CHS, Sigma) in PBS (10 μl of 6 μmol/ mL) – termed “CHS”. For each of these five conditions: 2 wells were exposed only the condition stated; 2 wells were pre-incubated for 24 h with rabbit anti-apolipoprotein B antibodies (anti-ApoB, 1 μL, abcam ab20737), blocking the receptor – termed “-ApoB”; 2 wells were also exposed to low density lipoprotein (LDL, 20 μg/ mL, Sigma) – termed “LDL”; 2 wells were also exposed to lipopolysaccharide (LPS, 0.4 μg/ mL, Sigma) – termed “LPS”; 2 wells were cultured in the presence of 10% FBS – termed “Ser”. Cells were incubated in each of these 25 different conditions for 24 h prior to either: fixing and staining for imaging; or performing the MTT viability assay.

### MTT assay

The MTT assay to measure the number of viable cells in each condition was performed according to manufacturer recommendation (V13154, Molecular Probes; 12 mM MTT incubated for 4 h, SDS-HCL incubated for 18 h) and absorbance was read using a multiplate reader (570 nm, Tecan Inifite 200 Pro). Absolute cell numbers were calculated by comparison with a calibration experiment conducted with the same THP-1 stock.

### Staining and microscopy

Cells were fixed for 20 min at room temperature with 2% paraformaldehyde. Following fixation and adequate washing using PBS, Nile red stain (1 μg/mL, 19,123, Sigma) was added to the wells and incubated at 37 °C for 10 min in the dark [7, 25]. The wells were then washed thrice using PBS and subsequently cell nuclei were counterstained with 4′,6-Diamidino-2-Phenylindole, Dihydrochloride (DAPI, D1306, Molecular Probes).The cells were imaged with a fluorescence microscope using the bright-field, DAPI and rhodamine filters (Axio Zeiss Observer Z.1, magnifications 20× and 40×, DAPI Ex/Em: 360/450 nm, Nile Red Ex/Em: 530/635 nm). Three images were taken per well at higher magnifications for qualitative analysis and one image was taken per well at 10× for quantitative image analysis.

### Image analysis

**Fig. 1:**
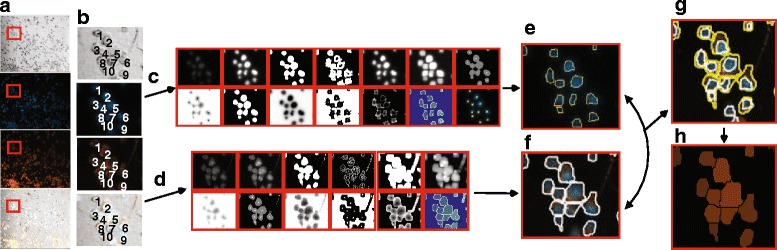
Algorithm scheme of automated macrophage/foam cell detection using a subset of 10 cells with condition phosphate buffered saline (PBS) control. Block **a**: Raw microscope images at 10× magnification: (top to bottom) Bright-field, Blue (DAPI-stained nucleus) channel, Red (Nile Red-stained lipids) channel, Blue + Red channel. Block **b**: sample of 10 cells are used to illustrate a (more) detailed outline of the image processing program. Block **c** includes the series of steps each blue channel image undergoes for (per cell) nucleus segmentation and identification. Block **d** includes the series of steps each ‘composite’ image undergoes, each red/blue region (cell) is identified based on the outlines of the red/blue regions. Block **e** shows the outlined nuclei that were identified within that image. Block **f** shows the outlines of the regions of interest (potential cells) which were identified within that image. Only such regions that contained one corresponding nucleus were accepted as cells. Block **h** average red intensities

**Fig. 2:**
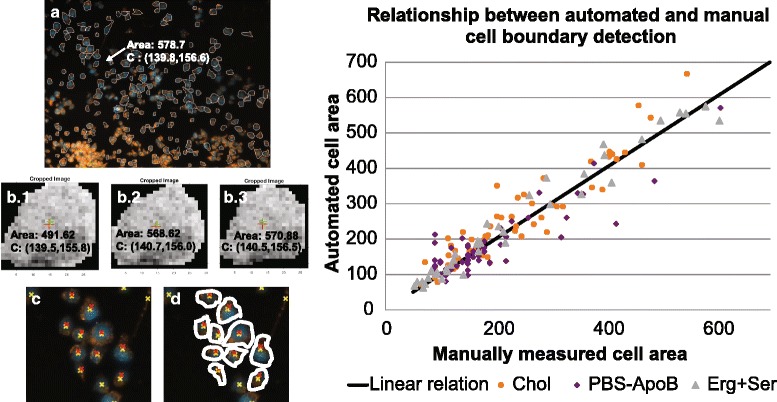
Comparison of automatically and manually measured cell boundaries for three conditions. **a** Raw image of merged red/blue channels with superimposed auto-generated cell boundaries (Condition: Chol), arrow identifies sample cell with automated area and centroid coordinates. **b** Shows manually measured output for one cell, traced three times, showing a range on area estimates, and little change in centroid location, the average area is treated as the manual area estimate for each cell. **c** Cropped portion of raw image with superimposed automatically (yellow) and manually (red) measured centroid estimates. **d** Sample of manually traced cell boundaries. (Graph) Corresponding manual area (a^) and automated (a*) are distributed around a 1:1 line of correspondence (161 data points). Absolute percent difference between (a^) and (a*) is on average 33%. Out of 161 manually measured cell boundaries, 95 (59%) area estimates measured below their automated counterpart and 66 (41%) over-estimated the area

Here, three superimposed images were recorded – a blue channel fluorescence image of DAPI-stained nuclei, a red channel fluorescence image of Nile Red-stained lipophilic material and a grey-scale bright-field image. All images were imported into MATLAB (The MathWorks Inc., MA, USA) for processing. First, the intensity and edges in the blue channel image were used to identify each nucleus. At the same time, the blue and red images were merged, and intensities and edges were similarly used to segment the merged image. Once these two steps were complete, the regions identified as nuclei from the blue image were mapped onto the merged image, and any segments in the merged image that either did not contain a nucleus, or that contained multiple nuclei, were excluded from analysis, leaving behind only segments identified as ‘cells’ (see Fig. 1). The resultant segmentation was compared to manually measured data from the associated bright-field image in order to assess the accuracy of this method (see Fig. 2). Additional comparison of algorithm effectiveness in different conditions is provided in Additional file 1: Figure S1.

The whole image analysis process is summarized in figure form in Fig. 1. The actual process of segmentation is conducted as follows: (1) image is converted to grey-scale, and contrast and intensity are enhanced to facilitate edge-detection; (2) edges are detected using Sobel, followed by dilation, filling of holes, erosion, removal of small objects and conversion to binary; (3) the resulting binary image is used as a mask for the grey-scale image; (4) contrast is enhanced in the masked grey-scale image and it is converted to binary following dilation and filling of holes; (5) this second binary image is reversed and the distance transformation of the image is calculated and negated; (6) the extended minima transform is computed (the regional minima of the H-minima transform connected by pixels having the same value – usually 0.6 for blue images and 1 or 2 for red-blue merged images) to remove regional minima that would cause over-segmentation in watershedding, resulting in an array termed X; (7) the intensity of the image with the negated distance transform is adjusted such that it only has regional minima wherever X is non-zero; (8) the complement of the binary image is computed so that the peaks become valleys, and this image is modified such that the background pixels and the extended maxima pixels are forced to be the only local minima by computing a logical array from the values in the reversed binary image and array X; (9) finally, watershedding is applied. Objects connected to the image boundary are neglected, and the following parameters are collected for each segment: centroid, area, perimeter, eccentricity, minor axis length and major axis length – of which area and eccentricity were explicitly analyzed in this work. Note that this strategy – designed to eliminate poorly resolved cell clusters – will also inevitably exclude multinuclear cells from analysis. As such cells comprise a fraction of macrophages in culture [26], this was considered an acceptable compromise to ensure that segmented images accurately identified single cells.

We replaced the real intensity with average per-cell intensities to qualitatively analyze cell-to-cell variations. Each cell’s average intensity is divided by brightest pixel intensity across all images to generate single intensity values for each cell.

### Statistical analysis

All statistical analysis was conducted in OriginPro software (OriginLab Corp. MA, USA) The appropriate hypothesis test was selected for each comparison as follows: if one or more datasets are not considered normally distributed, then a non-parametric Mann-Whitney test is used; if both datasets are considered normally distributed, and have equal variance, then a standard t-test is used; or, if both datasets are considered normally distributed but have unequal variances, then a Welch-corrected t-test is used. Normality and variance were assessed with *p* < 0.05. For the purpose of this study, a difference between two conditions was considered notable if it was significant at *p* < 0.001. Average (mean) values are quoted ± standard error of the mean (SEM). A full summary of all statistical comparisons for cell area and average red intensity data is included in the Additional file 1: Tables S1 to S3.

For linear correlations between red intensity and area: a correlation is considered significant if it is significant (*p* < 0.05) in both Pearson and Spearman tests. It is not considered significant if it is significant in Spearman but not Pearson (as this implies that there may be a non-linear correlation), and is not considered significant if it is not significant in either test, or is significant in Pearson but not Spearman (given the lack of normality of much of the data, and the presence of outliers, the Pearson test would not give a sufficiently reliable conclusion in this study).

## Results

All data sets were analyzed with the newly developed automatic method for segmentation of both cell nuclei and ‘composite’ cell boundaries. Each data set represents a different condition, resulting in a range of 40 – 1012 cells per condition. In all the data sets, the algorithm gave comparable results regardless of differences between images. Some images displayed sparsely distributed cells, while other images showed more cell clusters (see Additional file 1: Figure S1). The segmentation results (area and centroid) were also compared to their manually measured counterpart, by testing on three separate conditions, Chol, PBS ApoB, Erg + Ser (see Fig. 2).

### Effect of cholesterol on cell size and lipid content

**Fig. 3:**
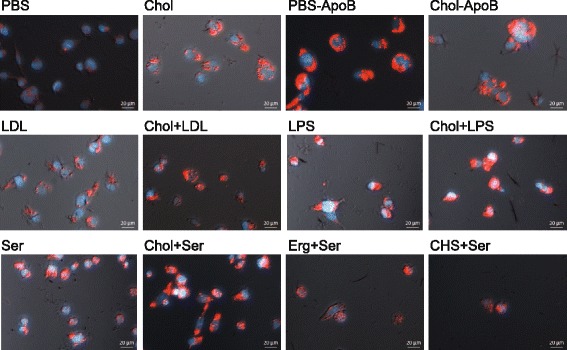
Two-channel fluorescence images of THP-1 derived macrophages exposed to various conditions. Nuclei were stained with DAPI (blue), lipids were stained with Nile Red (red), and cells were imaged at 20× magnification. Scale bar is 20 μm. -ApoB (pre-cultured with rabbit anti-apolipoprotein B antibodies), Chol (cholesterol and 1-palmitoyl-lyso-phosphatidylcholine (LPC) in PBS), CHS (cholesteryl hemisuccinate and LPC in PBS), Erg (ergosterol and LPC in PBS), LDL (low-density lipoprotein in PBS), LPS (lipopolysaccharide in PBS), PBS (phosphate-buffered saline vehicle), Ser (medium contained 10% fetal bovine serum)

**Fig. 4:**
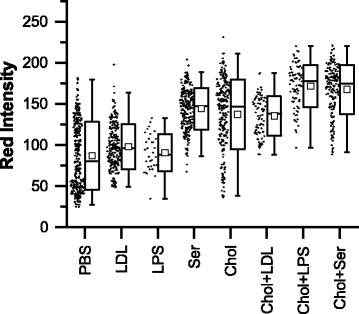
Summary of red intensity data for control samples and samples with cholesterol. Each point represents the average Nile Red fluorescence (i.e. red intensity normalized to maximum pixel intensity across all images) for one cell. In the box-and-whisker representation, the whiskers show the 1-99 percentiles, the box shows the standard deviation, the clear square shows the mean and the horizontal line shows the median. In all cases, the cholesterol condition had significantly (*p* < 0.001) increased red intensity compared with the paired control (i.e. Chol vs PBS or Chol + LDL vs LDL). Chol (cholesterol and 1-palmitoyl-lyso-phosphatidylcholine (LPC) in PBS), LDL (low-density lipoprotein in PBS), LPS (lipopolysaccharide in PBS), PBS (phosphate-buffered saline vehicle), Ser (medium contained 10% fetal bovine serum)

One easily observable feature of foam cells compared with their parent macrophages is the presence of lipid droplets, or increased lipid content, which can be visualized by staining with an appropriate lipophilic dye (Fig. 3) [25, 27]. An indirect measure of cell lipid content was made by quantifying the Nile Red fluorescence intensity (hereafter termed ‘red intensity’) per cell, as an average of individual pixel intensities within a particular cell border. Red intensity data for the control samples and those with cholesterol are shown in Fig. 4.

When comparing each cholesterol-containing sample with its paired control (i.e. PBS with Chol, LDL with Chol + LDL, LPS with Chol + LPS and Ser with Chol + Ser), it is found that in all cases there was a significant (*p* < 0.001) increase in average red intensity in the cholesterol containing samples compared with the relevant control. There is also a significant (*p* < 0.001) increase in the average red intensities in the control sample incubated with serum-containing medium (labeled Ser), compared with the control samples that were incubated in a serum-free environment (labeled PBS, LDL or LPS). Although less qualitatively apparent from the data plot (Fig. 4), there is a similarly significant (*p* < 0.001) although less substantial increase in the average red intensities in the sample containing cholesterol and serum, compared with the sample containing cholesterol or cholesterol with LDL in the absence of serum, although there is no difference between the samples containing cholesterol and LPS and the sample containing cholesterol and serum. The effect of LDL on increasing lipid content is less apparent; there is a slight but significant (*p* < 0.001) increase in red intensity in cells exposed to LDL versus the PBS control, and there is no difference in red intensity in cells exposed to both LDL and cholesterol compared with cells exposed to cholesterol alone. Similarly, in the absence of cholesterol, there was no significant effect of LPS on average intensity compared with the control sample (PBS), although there was an increase (*p* < 0.001) in average red intensity in samples cultured with both cholesterol and LPS, compared with samples cultured with cholesterol alone. The magnitude of the changes in Nile Red intensity between control and cholesterol-containing condition were quantified (e.g. from 87.0 ± 2.3 to 137.4 ± 2.8 au (increased by 58%) when comparing PBS to Chol + PBS, from 98.0 ± 1.8 to 135.5 ± 2.7 au (increased by 38%) when comparing LDL to Chol + LDL, and from 90.7 ± 4.0 to 171.7 ± 2.9 au (increased by 89%) when comparing LPS to Chol + LPS). The exception to this observation is the comparison in serum-containing samples, in which case the average Nile Red intensity in the cholesterol-exposed sample increased by less (from 144.1 ± 1.7 to 167.4 ± 2.3 au (increased by 16%) when comparing Ser with Chol + Ser). The standard deviations in red intensity were also calculated. Values for cells exposed to PBS or to cholesterol only were considerably larger (at 41.1 and 42.3 au for PBS and Chol, respectively), than the standard deviation for cells exposed to any other condition (between 22.6 and 29.9 au for LDL, Chol + LDL, LPS, Chol + LPS, Ser and Chol + Ser).

Area per cell was also calculated (see Additional file 1: Figure S2), in order to determine whether any conditions promoted cellular hypertrophy. Initial evidence suggests that the distribution of cell areas is less sensitive to condition than the average red intensity per cell. Average cell area varied between 84 ± 3 μm^2^ in the Chol + Ser sample and 121 ± 3 μm^2^ in the LDL sample, with large standard deviations reflecting considerable heterogeneity in cell size.

When comparing the effect on cell area of each cholesterol containing sample with its paired control (i.e. PBS with Chol, LDL with Chol + LDL, LPS with Chol + LPS and Ser with Chol + Ser), there was no significant effect of adding cholesterol compared with the PBS control (i.e. PBS vs Chol) or with the LPS control (i.e. LPS vs Chol + LPS), and there was a slight but significant (*p* < 0.001) decrease in area in samples containing cholesterol and LDL or serum compared with those containing LDL or serum alone. This was a somewhat unexpected result, so we also plotted red intensity versus area for each cell in each condition, in order to determine whether, under any conditions, there was a correlation between red intensity and cell size (See Additional file 1: Figure S3). There was a significant (*p* < 0.05) positive correlation between area and red intensity for the LDL (Pearson *R* = 0.27465, Spearman *R* = 0.26366), Chol + LPS (Pearson *R* = 0.30092, Spearman *R* = 0.295) and Chol + Ser (Pearson *R* = 0.15835, Spearman *R* = 0.29925) conditions, and a significant (*p* < 0.05) negative correlation for the PBS (Pearson *R* = −0.21734, Spearman *R* = −0.19991) and Chol (Pearson *R* = −0.36326, Spearman *R* = −0.26028) conditions, and possible positive correlations for the LPS (Spearman *R* = 0.41789) and Ser (Spearman *R* = 0.13701) conditions.

**Fig. 5:**
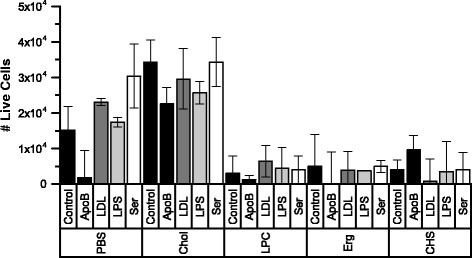
Results of MTT assay for all conditions. The MTT assay of the number of viable cells in each condition was carried out according to manufacturer instructions. Results were quantified with a multiplate reader and calibrated against standards generated from the same cell stock. -ApoB (pre-cultured with rabbit anti-apolipoprotein B antibodies), Chol (cholesterol and 1-palmitoyl-lyso-phosphatidylcholine (LPC) in PBS), CHS (cholesteryl hemisuccinate and LPC in PBS), Erg (ergosterol and LPC in PBS), LDL (low-density lipoprotein in PBS), LPC (LPC in PBS), LPS (lipopolysaccharide in PBS), PBS (phosphate-buffered saline vehicle), Ser (medium contained 10% fetal bovine serum)

In addition to image analysis, an MTT assay was conducted to assess cell survival at the experiment end point. The results of this assay are plotted in Fig. 5. In general, more cells survived in samples containing cholesterol than in the paired control.

Based on the analyses described herein, we conclude that the addition of cholesterol, particularly in combination with LPS, induces a significant increase in the lipid content of THP-1-derived macrophages compared with controls. Although no increase in cell size is seen overall in any cell population in a cholesterol-containing condition compared with its control, samples with the highest average red intensity per cell (i.e. Chol + LPS and Chol + Ser) also have a positive linear correlation between red intensity and cell area, indicating that in these conditions, as cells increase in lipid content, they also increase in size. The addition of cholesterol alone did not induce any positive correlation between area and red intensity, and scatter plots of red intensity versus area for both the control (PBS) and the cholesterol (Chol) samples (Additional file 1: Figure S3) show a large distribution of cell area and red intensity in these conditions, indicating that the cell population is diverse in these two parameters.

### Effect of sterol structure on cell size and lipid content

**Fig. 6:**
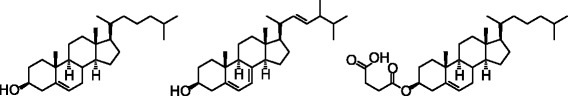
Molecular structures of selected sterols. Structures of cholesterol (left), ergosterol (center), and cholesteryl hemisuccinate (CHS, right)

In order to determine the molecular specificity of the effect of cholesterol on macrophages, we used four different lipid additives: cholesterol with LPC, as described with murine macrophages previously [10, 11]; LPC alone; ergosterol with LPC; and CHS with LPC (see Fig. 6). The LPC condition was selected to ensure that the observed effects were related to cholesterol, or to the particular combination of LPC with cholesterol, and not to the LPC carrier alone, as it has been previously reported that LPC itself contributes to macrophage activation [28].

**Fig. 7:**
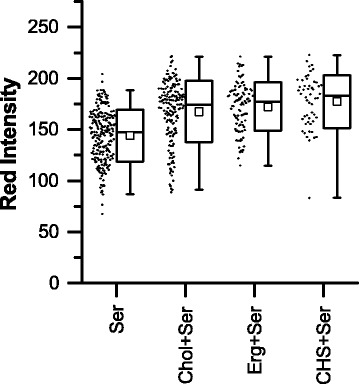
Summary of red intensity data for control samples and samples with different sterols. Each point represents the average Nile Red fluorescence for one cell (i.e. red intensity normalized to maximum pixel intensity across all images). In the box-and-whisker representation, the whiskers show the 1-99 percentiles, the box shows the standard deviation, the clear square shows the mean and the horizontal line shows the median. Chol (cholesterol and 1-palmitoyl-lyso-phosphatidylcholine (LPC) in PBS), CHS (cholesteryl hemisuccinate and LPC in PBS), Erg (ergosterol and LPC in PBS), Ser (medium contained 10% fetal bovine serum)

The LPC-only condition exhibited severe cellular toxicity within the 24-h experiment duration, with each well exposed to this condition having insufficient remaining cells for image analysis (see MTT results in Fig. 5). Under these experimental conditions, LPC forms a micellar solution. In comparison, LPC mixed in a 1:1 M ratio with cholesterol is likely in a lamellar phase, with the sample comprising a turbid suspension of multi-lamellar vesicles [29, 30]. The turbidity of the ergosterol and CHS samples indicates that these mixtures were also not comprised of mixed micelles, and no evidence of micelles was found in dynamic light scattering (DLS) experiments, with all of these samples apparently consisting of larger aggregates (data not shown). Both ergosterol and CHS were considerably more cytotoxic than cholesterol, with cell numbers reduced from the initially deposited 21,000 per well to less than 10,000 in conditions containing ergosterol or CHS, compared with final cell numbers above 20,000 for all conditions containing cholesterol (Fig. 5). Of all of the conditions containing ergosterol or CHS, useful images were only obtained for serum-containing wells (Erg + Ser and CHS + Ser), and these showed increased lipid content (Fig. 7) compared with the serum-only control (Ser). The increase in lipid content was similar in magnitude to the Chol + Ser condition. The red intensity and area data for cells exposed to cholesterol, ergosterol or CHS in the presence of serum are shown in Fig. 7 and Additional file 1: Figure S4.

In serum-containing wells, all sterol-containing conditions (i.e. Chol + Ser, Erg + Ser and CHS + Ser) exhibited significantly (*p* < 0.001) increased red intensity compared with the non-sterol control (i.e. Ser). However, there was no significant difference between red intensity distributions in cells cultured with cholesterol, ergosterol or CHS. Cell area was again less sensitive to the change in conditions, with the cholesterol and ergosterol conditions resulting in a small but significant (*p* < 0.001) decrease in average cell area compared with the control (i.e. Ser vs Chol + Ser and Ser vs Erg + Ser). There was no difference in the distribution of areas in the CHS condition compared with the control. Plots of red intensity versus area (Additional file 1: Figure S5) showed a significant (*p* < 0.05) positive correlation between cell size and lipid content only for the sample containing cholesterol, with a possible correlation between cell size and lipid content for the serum control.

Based on these analyses of cells exposed to different sterol conditions, we conclude that although all three of the sterols studied (cholesterol, ergosterol and CHS) induce an increase in cell lipid content in the presence of serum, only with cholesterol is this increased lipid content correlated with increased cell size, and sterols other than cholesterol exhibited significant toxicity.

### Effect of pre-incubation with anti-ApoB antibodies

**Fig. 8:**
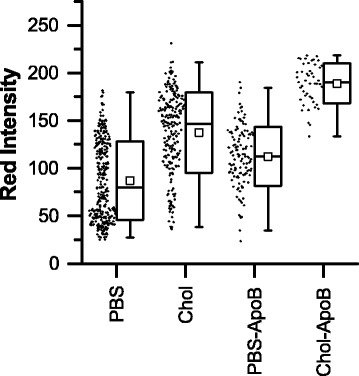
Summary of red intensity data for samples pre-incubated with or without anti-apolipoprotein B antibodies. Each point represents the average Nile Red fluorescence for one cell (i.e. red intensity normalized to maximum pixel intensity across all images). In the box-and-whisker representation, the whiskers show the 1-99 percentiles, the box shows the standard deviation, the clear square shows the mean and the horizontal line shows the median. -ApoB (pre-cultured with rabbit anti-apolipoprotein B antibodies), Chol (cholesterol and 1-palmitoyl-lyso-phosphatidylcholine (LPC) in PBS), PBS (phosphate-buffered saline vehicle)

The effect of pre-incubation with anti-ApoB antibodies on red intensity (Fig. 8) and area (Additional file 1: Figure S6) was measured in order to investigate a potential cellular mechanism via which the exogenous cholesterol may interact with the THP-1 macrophages. There was a slight increase in area and a somewhat larger increase in red intensity in cells pre-incubated with -ApoB (PBS-ApoB) compared with the control (PBS) (*p* < 0.001). For cells exposed to cholesterol, there was an increase in red intensity but no change in area in cells pre-incubated with ApoB antibodies compared with the control (Chol) (*p* < 0.001). Additional file 1: Figure S7 illustrates the relationship between cell area and red intensity for samples pre-incubated with anti-ApoB antibodies compared with controls. There was no significant linear correlation between area and red intensity in cholesterol-exposed cells pre-incubated with anti-ApoB (Pearson *R* = −0.06331, Spearman *R* = −0.11748). In control cells pre-incubated with anti-ApoB antibodies, there was a weak but significant (*p* < 0.05) positive linear correlation between area and red intensity (Pearson *R* = 0.19328, Spearman *R* = 0.24183). These are interesting observations, because in both the PBS control and the cholesterol control, there was a slight negative linear correlation between area and red intensity. Therefore, the anti-ApoB antibodies had some effect in increasing lipid content and altering the relationship between cell area and lipid content, and this effect was more pronounced in the control sample than in the sample exposed to cholesterol.

### Extraction of additional parameters by automated image analysis

**Fig. 9:**
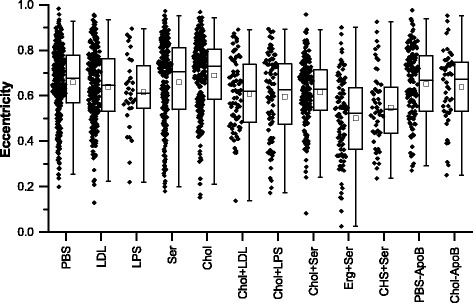
Cell eccentricity in different conditions. These eccentricity results report on how “round” each cell is, with a value of 0 for a circle and a value of 1 for an ellipse with a large long-axis:short-axis ratio. -ApoB (pre-cultured with rabbit anti-apolipoprotein B antibodies), Chol (cholesterol and 1-palmitoyl-lyso-phosphatidylcholine (LPC) in PBS), CHS (cholesteryl hemisuccinate and LPC in PBS), Erg (ergosterol and LPC in PBS), LDL (low-density lipoprotein in PBS), LPC (LPC in PBS), LPS (lipopolysaccharide in PBS), PBS (phosphate-buffered saline vehicle), Ser (medium contained 10% fetal bovine serum)

In addition to cell area and red intensity (as a measure of lipid content), automated image analysis can also be used to extract a variety of parameters, such as eccentricity, which is a measure of the roundness of each cell (Fig. 9). Average eccentricity ranged between 0.502 ± 0.018 for the Erg + Ser condition and 0.689 ± 0.011 for the Chol condition. In the absence of serum, there were no significant differences between paired conditions (i.e., PBS vs Chol, LDL vs Chol + LDL, LPS vs Chol + LPS, PBS vs PBS-ApoB, Chol vs Chol-ApoB or PBS-ApoB vs Chol-ApoB). However there were significant (*p* < 0.001) reductions in eccentricity in serum-containing conditions with sterols, compared with the serum-only control (i.e., Ser vs Chol + Ser, Ser vs Erg + Ser and Ser vs CHS + Ser), indicating that cells in these sterol-containing serum conditions exhibited somewhat altered morphology (increased roundness). In addition, cells in the ergosterol-containing serum condition exhibited a significantly (*p* < 0.001) reduced average eccentricity compared with the cholesterol-containing serum condition, whereas there was no such significant difference between the CHS-containing serum condition and the cholesterol-containing serum condition.

**Fig. 10:**
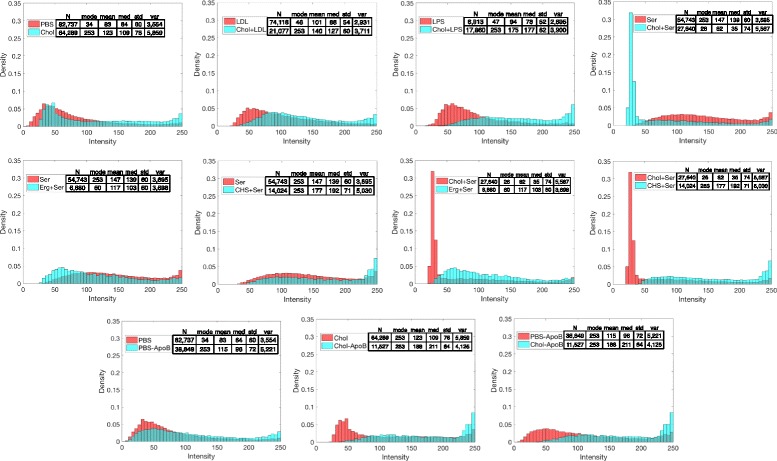
Distributions of pixel red intensity. These plots represent the distribution of pixels of different red intensity in identified cells within the stated conditions. Each condition contains a different number of cells, and cell size is also variable, therefore, the pixel intensity data will vary per condition. Each histogram has been normalized to represent ‘count-density’, rather than ‘frequency’ or ‘raw count’; the area of each bin represents the percent of observed pixels within that intensity range, rather than height. High-intensity pixels reflect a region with a high lipid concentration allowing dye to accumulate, suggestive of lipid bodies. -ApoB (pr We would interpret high-intensity pixels as reflecting an area with a high lipid concentration allowing dye to accumulate, suggestive of lipid bodies.e-cultured with rabbit anti-apolipoprotein B antibodies), Chol (cholesterol and 1-palmitoyl-lyso-phosphatidylcholine (LPC) in PBS), CHS (cholesteryl hemisuccinate and LPC in PBS), Erg (ergosterol and LPC in PBS), LDL (low-density lipoprotein in PBS), LPC (LPC in PBS), LPS (lipopolysaccharide in PBS), PBS (phosphate-buffered saline vehicle), Ser (medium contained 10% fetal bovine serum)

An examination of the distribution of red pixel intensities in different conditions may give some insight into if and how lipids and sterols are distributed within cells (Fig. 10). We would interpret high-intensity pixels as reflecting an area with a high lipid concentration allowing dye to accumulate, suggestive of lipid bodies. For example, the addition of cholesterol in comparison with control conditions generally increases the proportion of high-intensity pixels (e.g. for PBS vs Chol, LDL vs Chol + LDL and LPS vs Chol + LPS). In serum conditions, neither the addition of cholesterol nor the addition of ergosterol increase the proportion of high-intensity pixels compared with the serum control (e.g. Ser vs Chol + Ser and Ser vs Erg + Ser). However, the addition of CHS does increase the number of high-intensity pixels (e.g. Ser vs CHS + Ser). Pre-incubation with anti-ApoB antibodies also increases the proportion of high-intensity pixels in both control (PBS-ApoB) and cholesterol-containing condition (Chol-ApoB), but the change was more noticeable in the cholesterol-containing condition. Combined with the analysis of average red intensity data (Figs. 4, 7 and 8), these data are suggestive of differences in the manner in which lipid uptake is processed in different conditions.

## Discussion

Our segmentation strategy has several advantages: it is able to reveal subtle disparities in cell phenotype, images are processed automatically and rapidly, and cell boundaries are detected with consistent criteria. This also enables the generation of the massive quantities of physiological data necessary to identify important physiological features of cells and their respective conditions. Although several sophisticated cell segmentation methods exist [31–36], we present a new procedure that accurately segments macrophages under various conditions, including small clusters, without the need for high powered computing or any user-interaction (see reference [37], Figs. 1 and 2, and Additional file 1: Figure S1). When available, automated image processing has the potential to detect the causes of plaque formation, and improve diagnostics, thus providing prognostics for disease progression [38–40]. This new method has broad applicability and, as illustrated, can greatly expand the suite of quantifiable macrophage cell traits. Additional details and links to the MATLAB codes are available at the following URL (www.dropbox.com/sh/wrp9gysiqa3yzym/AAAw_-XYnmjJViLKf-5P2ikJa?dl=0). The main limitation of our image processing method in this study was in accurately identifying the boundaries of single cells within large clusters, and the algorithm parameters were set to ignore such clusters to prevent erroneous assignments. However, this limitation could easily be removed if cells were also stained with a suitable, commercially available plasma membrane dye and imaged in three channels (one for the Nile Red for general lipid content, one for the plasma membranes specifically and one for the DAPI-stained nuclei).

In agreement with previous studies in murine Raw 264.7 cells [10], and in THP-1 derived macrophages [11] we demonstrated that cholesterol-containing lipid particles induce increased lipid content in macrophages derived from human THP-1 cells. On visual inspection (Fig. 3), the appearance of the Nile Red lipid stain was similar to that reported for THP-1 macrophages exposed to oxLDL [7, 41]. When the intensity of the Nile Red stain per cell was quantified as an indirect measure of lipid content, cells exposed to cholesterol-containing lipid particles had increased lipid content compared with the negative control in all cases (Fig. 4). In previous studies, when compared to unexposed controls, total cholesterol content and total cholesterol ester content in THP-1 macrophages were found to increase by between ~50 and 200% on exposure to oxLDL [7, 41]. Total cholesterol and cholesterol esters in macrophages isolated from mice fed a high fat diet increased by similar amounts compared with mice on a normal diet [11]. With the exception of the serum condition, we observed increases in the average Nile Red intensity of a similar order of magnitude in samples containing cholesterol compared with their paired control. The increase in the serum condition was less, probably as the result of the increased intensity in the serum control due to the likely presence of lipids and other activating factors. Previous studies of these cholesterol-containing lipid particles with murine cells did not report the increase in cholesterol content relative to any control, but did show that the total cholesterol and cholesterol ester content of cells exposed to these particles was similar to that of cells exposed to acLDL [10]. Therefore, although our method of assessing lipid content via image analysis is indirect (i.e. measuring stain intensity, not extracting and measuring the target lipid directly from the whole cell population) and is not specific for cholesterol, our results for the average lipid content of the cell populations are comparable with other studies in similar systems, validating this approach.

The various controls and cholesterol-containing conditions studied had little impact on average cell area (Additional file 1: Figures S2, S4 and S6), in agreement with a previous report on foam cell formation in THP-1 macrophages that also used a 24-h incubation time [42], although there was a large standard deviation in area in many conditions. Large morphological variation in macrophage cultures has previously been reported for both primary-derived [26] and cell line-derived [42, 43] systems. It may be interesting in future work to examine the effects of prolonged incubation times with these different sterols, to determine the time-scales and eventual extent of the changes in these various cell properties.

The effect of exposing cells to unmodified LDL, and to LPS was also studied, as a control. Unmodified LDL is a form of additional negative control, as oxidized LDL (oxLDL) or acetylated LDL (acLDL) are more normally associated with the activation of macrophages to produce foam cells [44–46], and the effect of acLDL was previously studied in this system by Fernandez-Ruiz et al. [11]. The role of LPS in macrophage activation is complex [19], but LPS has been shown to prime macrophages for foam cell formation, by increasing triglyceride and cholesteryl ester uptake in the presence of lipid particles [47, 48]. Cells exposed to unmodified LDL showed no increase in lipid content compared with controls, and there was no synergistic effect of co-administering LDL with cholesterol (Fig. 4). This result is consistent with the generally accepted understanding that native (unmodified, not oxidized or acylated) LDL does not promote macrophage foam cell formation [44, 45], particularly at the relatively low concentrations (20 μg/mL) used in the present study [46]. The use of automated image analysis to further study the effects of LDL modification on foam cell formation could provide interesting single cell and cell population results in the future, particularly with reference to work such as that by Meyer et al. [49], who describe how the extent of LDL oxidation may affect macrophage response. Cells exposed to LPS in the absence of any additional source of exogenous lipid (i.e. the LPS condition without serum or any lipid particles), showed no increase in lipid content, but there was a small synergistic effect of LPS and cholesterol, with the Chol + LPS condition having a slight increase in lipid content compared with Chol, indicating some role for LPS-activated uptake of these particles.

Ergosterol and CHS were selected for this study to determine if any particular structural feature of the cholesterol molecule is vital for its activity. Ergosterol differs from cholesterol in its ring and hydrophobic chain region. In higher doses than used in this study (and in the absence of LPC), ergosterol reduced the pro-inflammatory effect of LPS-induced tumor necrosis factor α (TNF-α) production in murine macrophages over a 6-h period, without affecting cell viability [50]. Ergosterol peroxide has also been reported to downregulate LDLR expression in Raw 264.7 macrophages [50]. Our results for ergosterol indicate that this protective effect may be mitigated or even reversed if ergosterol is combined with other inflammatory lipids. In comparison, CHS differs from cholesterol in its polar head-group region, with the hydroxyl group being esterified, albeit with a much more polar group than the fatty-acid derived esters seen in endogenously produced cholesterol esters. CHS is not a naturally occurring compound, but has been investigated as a potential additive to liposome-based drug delivery systems. For example, it has been used in the preparation of pH sensitive liposomes, particularly for the delivery of oligonucleotides [51, 52]. The concentrations of sterol-containing lipid particles in our experimental conditions were ~1000 umol/ml – at which concentrations native LDL has been found to be taken up via receptor-independent pinocytotic pathways [49, 53]. Fluid-phase pinocytosis is known to be upregulated in human macrophages exposed to PMA [53, 54], and to contribute to acLDL uptake in THP-1 derived macrophages [55]. Similarly, uptake of free cholesterol from native LDL particles occurs mostly via whole-particle uptake rather than selective sterol uptake [46]. The lack of distinction between uptake of cholesterol, ergosterol and CHS suggests that a non-selective uptake pathway, such as pinocytosis, may be involved in lipid accumulation under these conditions, with the subsequent effect of the various internalized sterols varying, depending on the sterol structure – resulting in different cytotoxicity (see Fig. 5), morphological changes (see Fig. 9) and lipid distribution (see Fig. 10). The LDLR gene has been reported to be downregulated in macrophages exposed to either cholesterol or cholesterol ester containing particles, compared with the control, whereas ABCA1 and ABCG1 gene expression was upregulated and reduced HDL efflux was reduced on exposure to cholesterol compared with cholesterol esters [11].

Selected conditions were also pre-incubated with anti-ApoB antibodies to investigate whether the sterol-containing lipid particles were hijacking ApoB-dependent uptake, or were entering cells via another mechanism. Pre-incubation of cells with the anti-ApoB antibody resulted in an increase in lipid content with respect to the control in cells incubated with PBS only, or in cells incubated with cholesterol-containing lipid particles (Fig 8). This was a somewhat unexpected result, as these conditions did not include addition of any ApoB-containing substance. However, activation of the LXR receptor is known to inhibit native LDL uptake by pinocytosis [56], and LXR is known to be activated by oxysterols (either exogenously absorbed from oxLDL, or endogenously synthesized [57, 58]). Prior to these experiments, our cell stock was cultured in FBS, which is known to contain low levels of LDL that is capable of binding to human LDL receptors [59]. It is also known that THP-1 cells, specifically, are sensitive to the presence of ApoB [60]. Therefore it is possible that in our system, pre-incubation with anti-ApoB removed any residual LDL or oxLDL from serum that remained in culture following differentiation, thus removing a source of LXR activation and thereby increasing constitutive pinocytosis. This could increase non-selective uptake of fatty particles, potentially exacerbating foam cell formation. Further antibody studies should be conducted to more conclusively confirm the meaning of these results.

## Conclusion

We have used automated analysis of fluorescence images of THP-1-derived macrophages to compare the co-administration of cholesterol, ergosterol and CHS with various other conditions relevant to foam cell formation, including LDL, LPS and anti-ApoB antibodies. The use of automated image processing techniques to rapidly identify and characterize cells was particularly helpful in this study, as it enabled the efficient acquisition of data for many more individual cells than is usually feasible with manual measurements. Significant (*p* < 0.001) differences in both population averages and distributions of properties were observed for red intensity (a measure of lipid uptake) and eccentricity in certain conditions, and there were noticeable differences in cell survival, although no significant differences in distributions of cell area were observed. Notably, cholesterol, ergosterol and CHS all caused increases in cell lipid content, although this was accompanied by increased cytotoxicity in the case of CHS and ergosterol. There was a synergistic effect of cholesterol and LPS, in increasing lipid uptake and a similar effect was seen with cholesterol in cells pre-incubated with anti-ApoB antibodies. These observations add to our understanding of the complex, multifactorial nature of foam cell formation, and such studies may be extended in the future to examine how variations in initial conditions of foam cell formation impact the progression of atherosclerotic plaque formation in more complex systems in both models and in patients.

## Additional file

Additional file 1: Supplementary data.
**Figure S1**. Reliability of the image processing algorithm in different conditions. **Figure S2**. Summary of cell area data for control samples and samples with cholesterol. **Figure S3.** Scatter plots of average red intensity per cell versus cell area for control samples and samples with cholesterol. **Figure S4**. Summary of cell area data for control samples and samples with different sterols. **Figure S5**. Scatter plots of average red intensity per cell versus cell area for control samples and samples with different sterols. **Figure S6**. Summary of cell area data for samples pre-incubated with or without anti-apolipoprotein B antibodies. **Figure S7**. Scatter plots of average red intensity per cell versus cell area for samples pre-incubated with or without anti-apolipoprotein B antibodies. **Table S1**. Summary of statistical test results for control samples and samples with cholesterol. **Table S2**. Summary of statistical test results for control samples and samples with different sterols. **Table S3**. Summary of statistical test results for samples pre-incubated with or without anti-apolipoprotein B antibodies. (DOCX 2455 kb)

## Abbreviations

acLDL: Acylated low-density lipoprotein
ApoB: Apolipoprotein B
CHS: Cholesteryl hemisuccinate
DLS: Dynamic light scattering
LPC: 16:0 lysophosphatidylcholine
LPS: Lipopolysaccharide
LXR: Liver X receptor
oxLDL: Oxidized low-density lipoprotein
RXR: Retinoid X receptor
SRA: Scavenger receptor A
SREBP: Sterol regulatory element-binding protein
SOAT: Sterol O-acyltransferase
TNF-α: Tumor necrosis factor α
