# Supplementary material for: Quantification of sterol-specific response in human macrophages using automated imaged-based analysis
Source: Lipids Health Dis. 2017 Dec 13;16:242. doi: 10.1186/s12944-017-0629-9 (PMC5729278; doi:10.1186/s12944-017-0629-9)
Supplement: Additional file 1: Supplementary data. — Figure S1. Reliability of the image processing algorithm in different conditions. Figure S2. Summary of cell area data for control samples and samples with cholesterol. Figure S3. Scatter plots of average red intensity per cell versus cell area for control samples and samples with cholesterol. Figure S4. Summary of cell area data for control samples and samples with different sterols. Figure S5. Scatter plots of average red intensity per cell versus cell area for control samples and samples with different sterols. Figure S6. Summary of cell area data for samples pre-incubated with or without anti-apolipoprotein B antibodies. Figure S7. Scatter plots of average red intensity per cell versus cell area for samples pre-incubated with or without anti-apolipoprotein B antibodies. Table S1. Summary of statistical test results for control samples and samples with cholesterol. Table S2. Summary of statistical test results for control samples and samples with different sterols. Table S3. Summary of statistical test results for samples pre-incubated with or without anti-apolipoprotein B antibodies. (DOCX 2455 kb) [file 12944_2017_629_MOESM1_ESM.docx]

***Title:* Sterol-specific responses in human macrophages**

***Authors:*** ^1^Deborah L. Gater, ^2^Namareq Widatalla, ^2,3^Kinza Islam, ^2^Maryam AlRaeesi, ^2^Jeremy C.M. Teo, ^1,4^Yanthe E. Pearson

***Table of contents:***

| Supplemental Figure S1: Reliability of the image processing algorithm in different conditions | Page S2 |
| --- | --- |
| Supplemental Figure S2: Summary of cell area data for control samples and samples with cholesterol | Page S3 |
| Supplemental Figure S3: Scatter plots of average red intensity per cell versus cell area for control samples and samples with cholesterol | Page S4 |
| Supplemental Figure S4: Summary of cell area data for control samples and samples with different sterols | Page S5 |
| Supplemental Figure S5: Scatter plots of average red intensity per cell versus cell area for control samples and samples with different sterols | Page S6 |
| Supplemental Figure S6: Summary of cell area data for samples pre-incubated with or without anti-apolipoprotein B antibodies | Page S7 |
| Supplemental Figure S7: Scatter plots of average red intensity per cell versus cell area for samples pre-incubated with or without anti-apolipoprotein B antibodies | Page S8 |
| Supplemental Table S1: Summary of statistical test results for control samples and samples with cholesterol | Page S9 |
| Supplemental Table S2: Summary of statistical test results for control samples and samples with different sterols | Page S9 |
| Supplemental Table S3: Summary of statistical test results for samples pre-incubated with or without anti-apolipoprotein B antibodies | Page S10 |

**Supplementary Figure S1: Reliability of the image processing algorithm in different conditions**


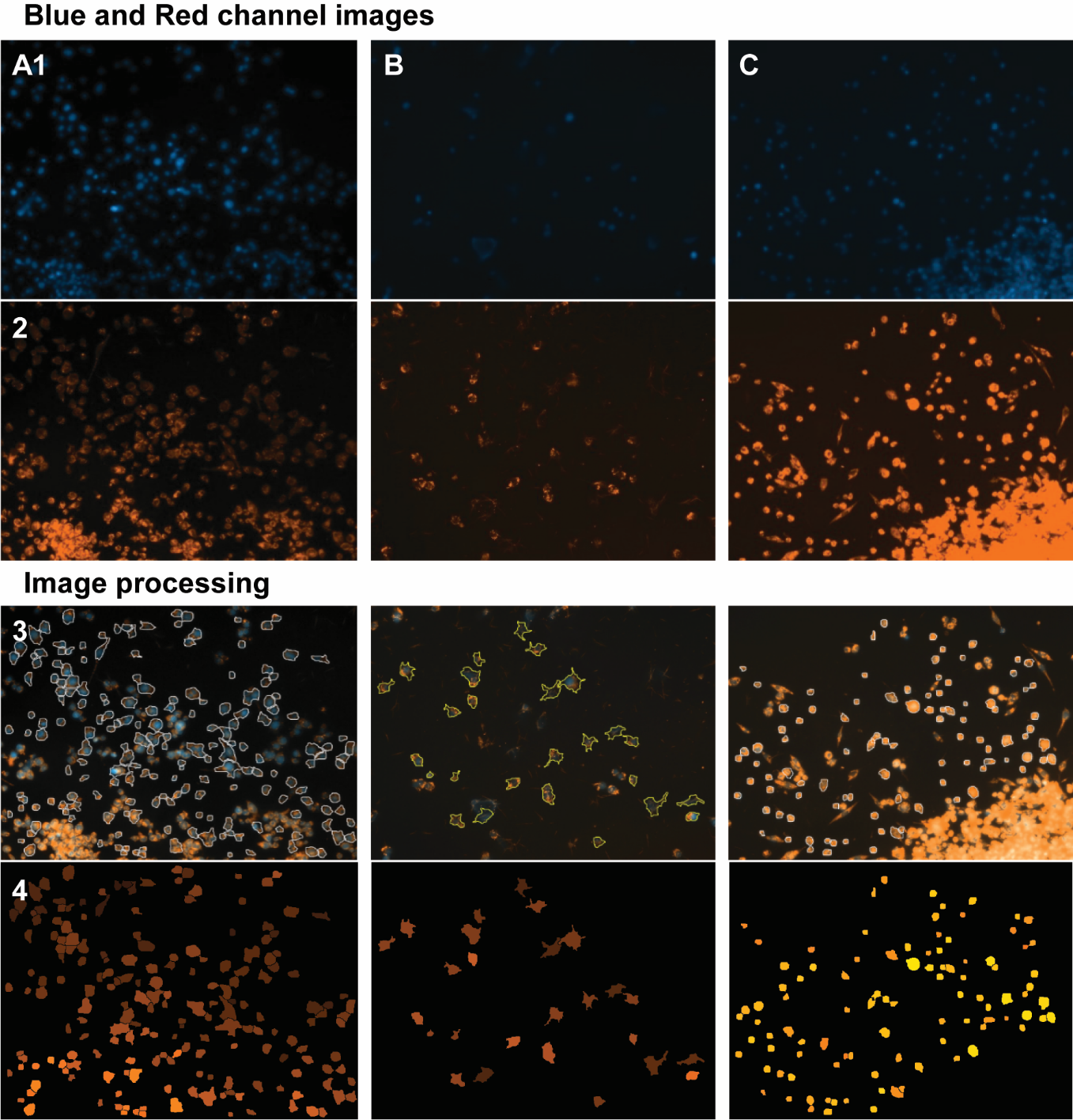


Here, we show step-wise algorithm output for three different conditions with dissimilar images. Robustness of the algorithm is measured by comparing manual and automated cell area and centroid. **Column A:** phosphate buffered saline (PBS) control, **Column B:** contains lyso-phosphatidylcholine (LPC) and cholesterol, and **Column C: contains** LPC, ergosterol and serum.  **Rows 1 & 2:** blue and red channels of the original image, respectively, **Row 4:** image cell borders (cells were selected based on common appearance in blue and original images). Our algorithm uses the image with both blue and red channels combined, due to difficulties with segmenting the original image. **Row 5:** each segmented region identified as an individual cell, with normalized red intensity.

**Supplementary Figure S2:** **Summary of cell area data for control samples and samples with cholesterol**


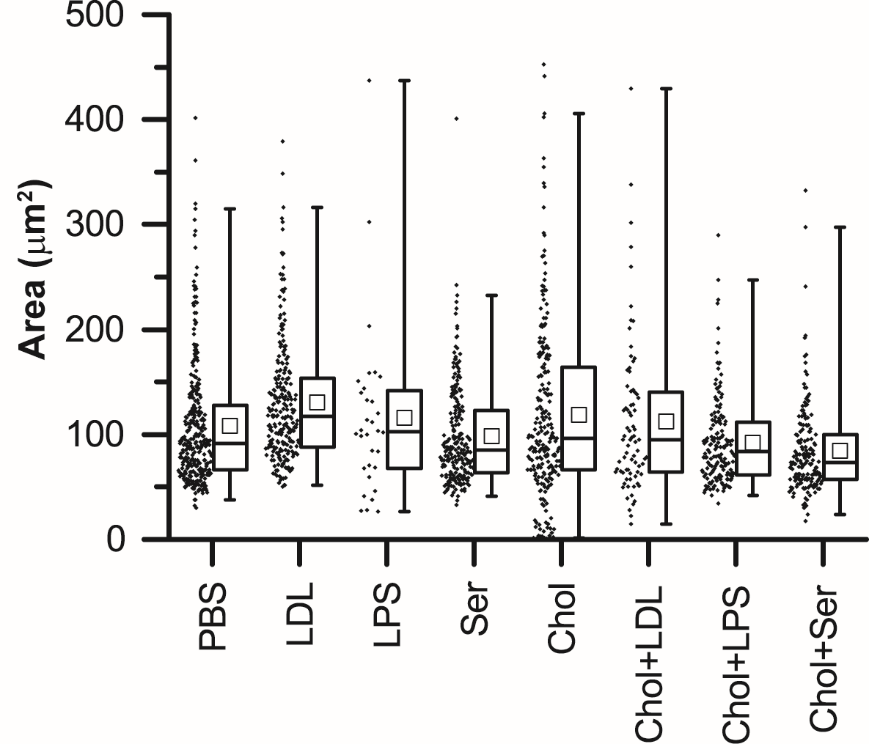


Each point represents the area (in μm^2^) of one cell. In the box-and-whisker representation, the whiskers show the 1-99 percentiles, the box shows the standard deviation, the clear square shows the mean and the horizontal line shows the median.

Key: **Chol** (cholesterol and 1-palmitoyl-lyso-phosphatidylcholine (LPC) in PBS), **LDL** (low-density lipoprotein in PBS), **LPS** (lipopolysaccharide in PBS), **PBS** (phosphate-buffered saline vehicle), **Ser** (medium contained 10% fetal bovine serum).

**Supplementary Figure S3: Scatter plots of average red intensity per cell versus cell area for control samples and samples with cholesterol**


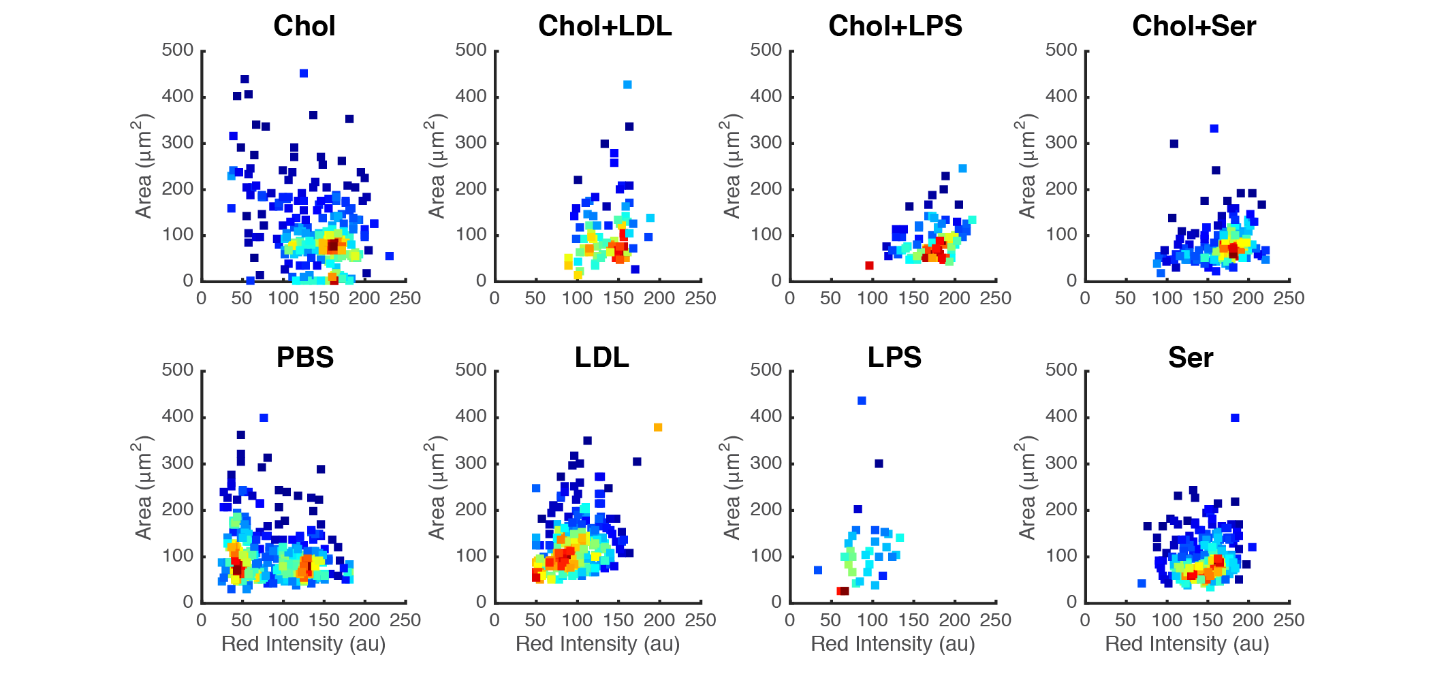


Each plot represents one culture condition, as labeled. Each point in each plot represents average Nile Red fluorescence vs area (in μm^2^).

Key: **Chol** (cholesterol and 1-palmitoyl-lyso-phosphatidylcholine (LPC) in PBS), **LDL** (low-density lipoprotein in PBS), **LPS** (lipopolysaccharide in PBS), **PBS** (phosphate-buffered saline vehicle), **Ser** (medium contained 10% fetal bovine serum).

**Supplementary Figure S4:** **Summary of cell area data for control samples and samples with different sterols**


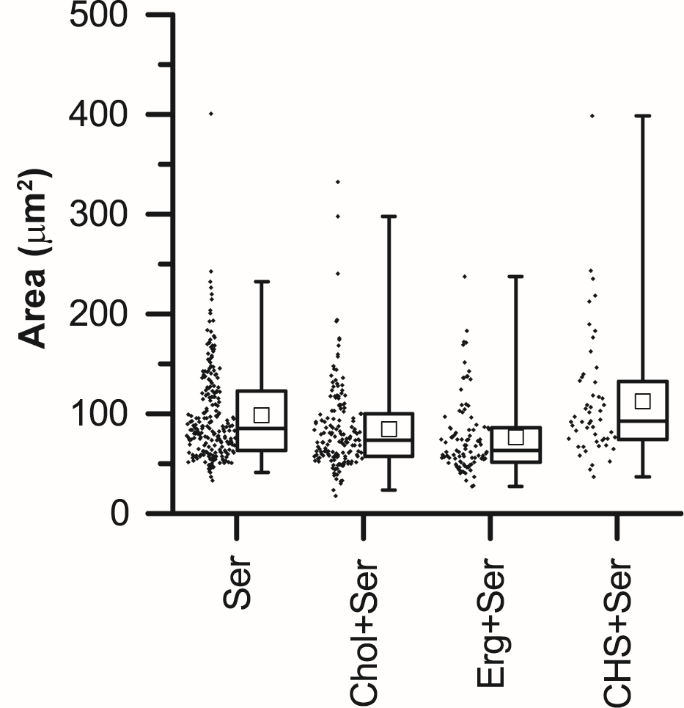


Each point represents the area (in μm^2^) of one cell. In the box-and-whisker representation, the whiskers show the 1-99 percentiles, the box shows the standard deviation, the clear square shows the mean and the horizontal line shows the median.

Key: **Chol** (cholesterol and 1-palmitoyl-lyso-phosphatidylcholine (LPC) in PBS), **CHS** (cholesteryl hemisuccinate and LPC in PBS), **Erg** (ergosterol and LPC in PBS), **Ser** (medium contained 10% fetal bovine serum).

**Supplementary Figure S5: Scatter plots of average red intensity per cell versus cell area for control samples and samples with different sterols**


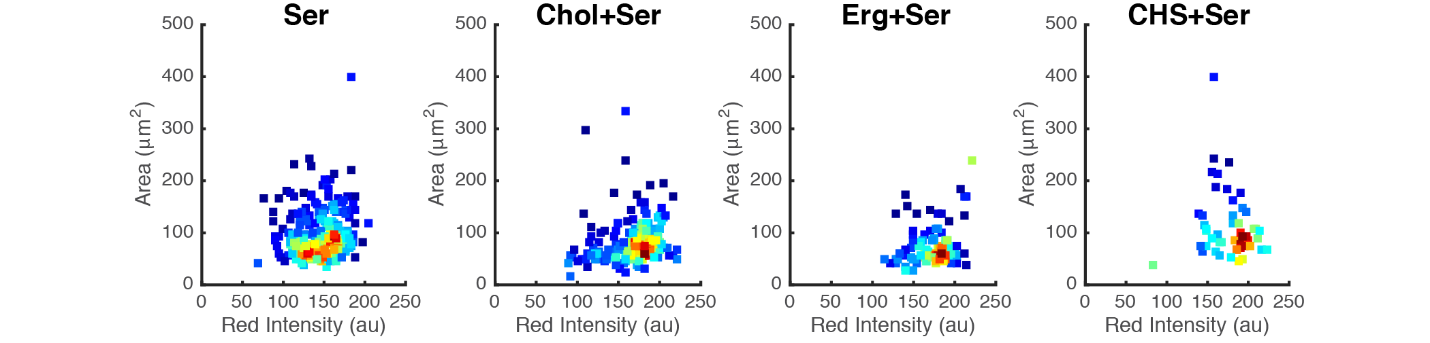


Each plot represents one culture condition, as labeled. Each point in each plot represents average Nile Red fluorescence vs area (in μm^2^).

Key: **Chol** (cholesterol and 1-palmitoyl-lyso-phosphatidylcholine (LPC) in PBS), **CHS** (cholesteryl hemisuccinate and LPC in PBS), **Erg** (ergosterol and LPC in PBS), **Ser** (medium contained 10% fetal bovine serum).

**Supplementary Figure S6: Summary of cell area data for samples pre-incubated with or without anti-apolipoprotein B antibodies**


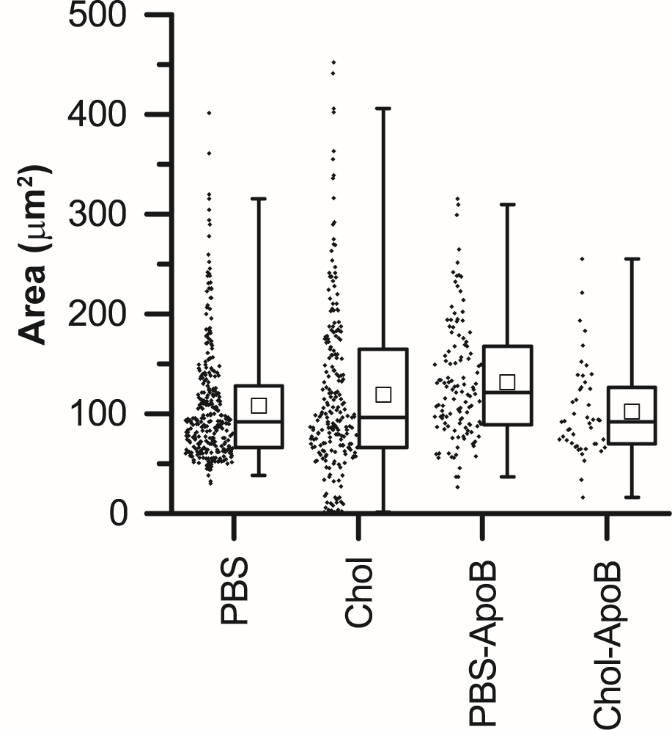


Each point represents the area (in μm^2^) of one cell. In the box-and-whisker representation, the whiskers show the 1-99 percentiles, the box shows the standard deviation, the clear square shows the mean and the horizontal line shows the median.

Key: **-ApoB** (pre-cultured with rabbit anti-apolipoprotein B antibodies), **Chol** (cholesterol and 1-palmitoyl-lyso-phosphatidylcholine (LPC) in PBS), **PBS** (phosphate-buffered saline vehicle).

**Supplementary Figure S7: Scatter plots of average red intensity per cell versus cell area for samples pre-incubated with or without anti-apolipoprotein B antibodies**


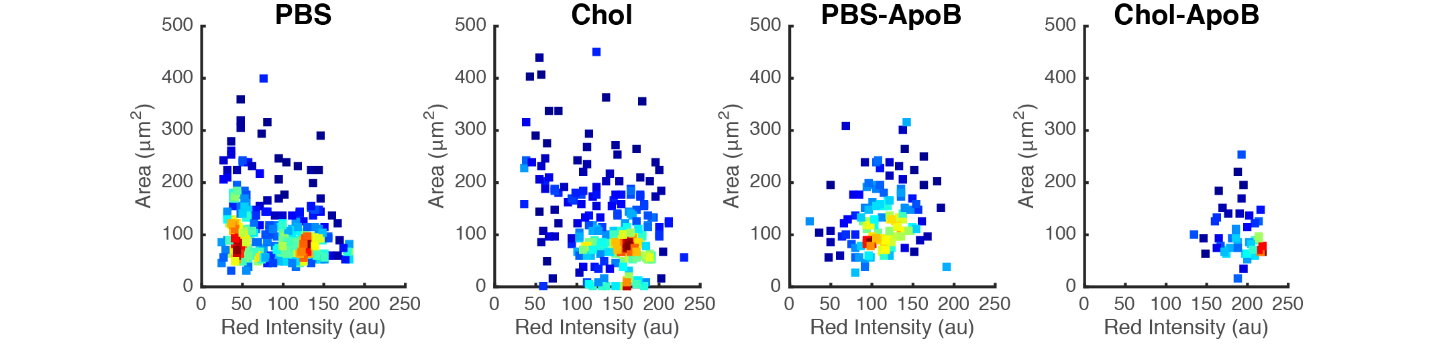


Each plot represents one culture condition, as labeled. Each point in each plot represents average Nile Red fluorescence vs area (in μm^2^).

Key: **-ApoB** (pre-cultured with rabbit anti-apolipoprotein B antibodies), **Chol** (cholesterol and 1-palmitoyl-lyso-phosphatidylcholine (LPC) in PBS), **PBS** (phosphate-buffered saline vehicle).

**Supplementary Table 1: Summary of statistical test results for control samples and samples with cholesterol**

|  | **PBS** | **LDL** | **LPS** | **Ser** | **Chol** | **Chol+LDL** | **Chol+LPS** | **Chol+Ser** |  |
| --- | --- | --- | --- | --- | --- | --- | --- | --- | --- |
| **PBS** |  | MW* | MW-o | MW* | MW* | MW* | MW* | MW* | RED INTENSITY |
| **LDL** | MW* |  | MW-o | MW* | MW* | MW* | MW* | MW* |  |
| **LPS** | MW-o | MW-o |  | T* | MW* | MW* | T* | MW* |  |
| **Ser** | MW-o | MW* | MW-o |  | MW-o | MW-o | T* | MW* |  |
| **Chol** | MW-o | MW* | MW-o | MW-o |  | MW-o | MW* | MW* |  |
| **Chol+LDL** | MW-o | MW* | MW-o | MW-o | MW* |  | MW* | MW* |  |
| **Chol+LPS** | MW-o | MW* | MW-o | MW-o | MW-o | MW-o |  | MW-o |  |
| **Chol+Ser** | MW* | MW* | MW-o | MW* | MW* | MW-o | MW-o |  |  |
|  | AREA | | | | | | |  |  |

Key: **MW** Mann-Whitney test due to one or more datasets for which normality was rejected with p < 0.05, **T** T-test for datasets for which normality could not be rejected with p < 0.05, ***** difference was significant at p < 0.001, **–o** difference was not significant at p < 0.001, **Chol** (cholesterol and 1-palmitoyl-lyso-phosphatidylcholine (LPC) in PBS), **LDL** (low-density lipoprotein in PBS), **LPS** (lipopolysaccharide in PBS), **PBS** (phosphate-buffered saline vehicle), **Ser** (medium contained 10% fetal bovine serum).

**Supplementary Table 2: Summary of statistical test results for control samples and samples with different sterols**

|  | **Ser** | **Chol+Ser** | **Erg+Ser** | **CHS+Ser** |  |
| --- | --- | --- | --- | --- | --- |
| **Ser** |  | MW* | T* | MW* | RED INT. |
| **Chol+Ser** | MW* |  | MW-o | MW-o |  |
| **Erg+Ser** | MW* | MW-o |  | MW-o |  |
| **CHS+Ser** | MW-o | MW* | MW* |  |  |
|  | AREA | | |  |  |

Key: **MW** Mann-Whitney test due to one or more datasets for which normality was rejected with p < 0.05, **T** T-test for datasets for which normality could not be rejected with p < 0.05, ***** difference was significant at p < 0.001, **–o** difference was not significant at p < 0.001, **Chol** (cholesterol and 1-palmitoyl-lyso-phosphatidylcholine (LPC) in PBS), **CHS** (cholesteryl hemisuccinate and LPC in PBS), **Erg** (ergosterol and LPC in PBS), **Ser** (medium contained 10% fetal bovine serum).

**Supplementary Table 3: Summary of statistical test results for samples pre-incubated with or without anti-apolipoprotein B antibodies**

|  | **PBS** | **Chol** | **PBS-ApoB** | **Chol-ApoB** |  |
| --- | --- | --- | --- | --- | --- |
| **PBS** |  | MW* | MW* | MW* | RED INT. |
| **Chol** | MW-o |  | MW* | MW* |  |
| **PBS-ApoB** | MW* | MW-o |  | MW* |  |
| **Chol-ApoB** | MW-o | MW-o | MW-o |  |  |
|  | AREA | | |  |  |

Key: **MW** Mann-Whitney test due to one or more datasets for which normality was rejected with p < 0.05, **T** T-test for datasets for which normality could not be rejected with p < 0.05, ***** difference was significant at p < 0.001, **–o** difference was not significant at p < 0.001, **-ApoB** (pre-cultured with rabbit anti-apolipoprotein B antibodies), **Chol** (cholesterol and 1-palmitoyl-lyso-phosphatidylcholine (LPC) in PBS), **PBS** (phosphate-buffered saline vehicle).
